# The failure of drug repurposing for COVID-19 as an effect of excessive hypothesis testing and weak mechanistic evidence

**DOI:** 10.1007/s40656-022-00532-9

**Published:** 2022-10-18

**Authors:** Mariusz Maziarz, Adrian Stencel

**Affiliations:** 1 Interdisciplinary Centre for Ethics, Jagiellonian University, Grodzka 52, Kraków, Poland; 2 Institute of Philosophy, Jagiellonian University, Grodzka 52, Kraków, Poland

**Keywords:** Medical nihilism, Mechanistic evidence, EBM+, Excessive hypothesis testing, False-positive results, Covid-19

## Abstract

The current strategy of searching for an effective treatment for COVID-19 relies mainly on repurposing existing therapies developed to target other diseases. Conflicting results have emerged in regard to the efficacy of several tested compounds but later results were negative. The number of conducted and ongoing trials and the urgent need for a treatment pose the risk that false-positive results will be incorrectly interpreted as evidence for treatments’ efficacy and a ground for drug approval. Our purpose is twofold. First, we show that the number of drug-repurposing trials can explain the false-positive results. Second, we assess the evidence for treatments’ efficacy from the perspective of evidential pluralism and argue that considering mechanistic evidence is particularly needed in cases when the evidence from clinical trials is conflicting or of low quality. Our analysis is an application of the program of Evidence Based Medicine Plus (EBM+) to the drug repurposing trials for COVID. Our study shows that if decision-makers applied EBM+, authorizing the use of ineffective treatments would be less likely. We analyze the example of trials assessing the efficacy of hydroxychloroquine as a treatment for COVID-19 and mechanistic evidence in favor of and against its therapeutic power to draw a lesson for decision-makers and drug agencies on how excessive hypothesis testing can lead to spurious findings and how studying negative mechanistic evidence can be helpful in discriminating genuine from spurious results.

## Introduction

SARS-CoV-2 belongs to a family of human coronaviruses that cause the common cold and more severe conditions such as breathing difficulty and acute respiratory distress syndrome (ARDS) (Gaunt et al., 2010). Direct mortality of COVID-19, the disease caused by SARS-CoV-2, is not the only reason why the pandemic creates an unprecedented threat to the healthcare systems. Some patients require hospitalization (oxygen therapy and intensive care, in particular) (Alexandrova et al., 2021). Furthermore, the patients who recovered from COVID-19 might experience long-term cardiovascular and neurological consequences (Iadecola et al., 2020). As the SARS-CoV-2 pandemic is a global threat that affects millions of people, medical researchers are trying to develop a therapy for COVID-19 rapidly. Their efforts rely primarily on drug repurposing, i.e., identifying those existing or investigational drugs that are effective for COVID-19 (Parvathaneni & Gupta, 2020; Senanayake, 2020). The primary advantages of drug repurposing (as opposed to developing new compounds) are a shorter time of the process and a known risk profile of the existing drugs (Pushpakom et al., 2019).

The hope of this strategy is to find a therapy lowering mortality, alleviating symptoms, and/or shortening the course of the disease. Within the first 100 days of the pandemic, 689 randomized controlled trials (RCTs) have been conducted, ongoing, or in preparation (of which more than a 100 tested the efficacy of antimalarial drugs) (Janiaud et al., 2020). The number of trials is steadily growing. When the work on the manuscript has started (December 23rd, 2020), Clinicaltrials.gov listed as many as 4118 active studies targeting COVID-19, including 2302 randomized controlled trials (RCTs). Among over two thousand RCTs, 168 active or planned trials test hydroxychloroquine’s efficacy (HCQ) and 55—remdesivir. We excluded the studies that are terminated, suspended, or withdrawn. The current strategy of drug repurposing is clearly very tempting as it might accelerate the process of discovering the cure. However, as we delineate below, we are concerned that it is likely to result in many false-positive results (i.e., reports showing statistically significant differences between the treatment and control arms due to chance alone). In effect, several small studies of the same compound are likely to deliver inconsistent results. Conflicting evidence has already emerged in regard to the efficacy of remdesivir (Jiang et al., 2021), hydroxychloroquine (Lauriola et al., 2020), and convalescent plasma (Valk et al., 2020). The chronological order of results, where (false) positive results were followed by negative outcomes (e.g., WHO Solidarity Trial Consortium, 2021), has driven changes in treatment recommendations (Godlee, 2020; Sanders et al., 2020).

Such changes are concerning as they can undermine trust in the drug agencies’ expertise and give false hopes for effective treatments that trigger doubt in effective drugs and even could fuel anti-vaccine sentiment as people might become more skeptical toward modern medicine. Even though, under the usual circumstances, suspending its decision until larger and more conclusive trials or meta-analysis of several underpowered RCTs deliver more trustworthy evidence saves drug agencies from accepting and later discouraging pharmaceutical therapies, the SARS-CoV-2 pandemic, being an unprecedented public health emergency, requires rapid decision-making in far from ideal circumstances. This motivates our paper. We use the example of trials repurposing hydroxychloroquine (HCQ) as treatments for COVID-19 to argue that false-positive results should be expected to emerge from the vast number of repurposing trials and argue that using mechanistic evidence from laboratory and animal studies can help in resolving inconsistencies in the results of clinical trials and telling genuine from spurious results. The purpose of our case study is twofold. First, we offer an explanation of why clinical trials assessing the efficacy of repurposing candidates reported inconsistent results, which can also deepen our understanding of conflicting evidence delivered by normal science. Second, we support applying the approach of Evidence Based Medicine Plus (EBM+) to asses clinical evidence and argue that it is particularly useful when the results of clinical trials are conflicting or of low quality, but decisions nevertheless need to be made.

The approach of EBM+ can be considered as an extension to the standard view on assessing medical evidence known as evidence-based medicine (EBM) (see Sackett et al., 2000; Worrall, 2010). This view has led to developing evidence hierarchies (e.g., The Oxford Centre for Evidence Based Medicine (2009) that prioritize randomized-controlled trials (RCTs) and systematic reviews of RCTs over observational human studies, animal and in vitro research, and theories and expert opinion. Despite mechanistic evidence enters the EBM hierarchy informally, at the stage of developing new drugs and designing clinical trials (Andersen, 2012; Rocca, 2018), mechanisms are not considered explicitly when efficacy claims are evaluated, and if they are, mechanistic evidence is believed to be of lower quality in comparison to associational studies. This view results from prioritizing those research methods that deliver evidence less susceptible to bias or confounding (Borgerson, 2009; La Caze, 2009). The voices advising expanding the evidentiary base of the EBM movement (Anjum et al., 2020; Buetow & Kenealy, 2000; Clarke et al., 2013, 2014) and the Russo-Williamson Thesis (Russo & Williamson, 2007) requiring both difference-making and mechanistic evidence for establishing causality have motivated the emergence of the EBM+ movement (Parkkinen et al., 2018). The debate between the supporters of EBM and EBM+ is still ongoing (e.g., Howick, 2011; Canali, 2019; Williamson, 2019), and we believe that applying the latter approach to assessing the efficacy of repurposing candidates can not only be useful for improving the accuracy of therapeutic decisions but also support EBM+ as a program suitable for resolving empirical controversies resulting from inconsistent results of clinical trials. According to EBM+, “[a] well-established mechanism of action can support the efficacy claim, while a hypothesized mechanism that has little evidence or contrary evidence (i.e., lack of biological plausibility) can undermine the efficacy claim” (Aronson et al., 2021). Therefore, paying attention to mechanistic evidence can deliver additional ground to decide which of inconsistent results reported by the COVID-19 repurposing trials is likely to be accurate.

The structure of the paper is as follows. In Sect. 2, we argue that base-rate fallacy may account for the number of false-positive results that emerged in the field of drug repurposing for COVID-19. First, we apply statistical arguments regarding spurious findings due to multiple comparisons to the field of COVID-19 drug-repurposing trials. Second, we review the clinical literature assessing the efficacy of HCQ as a treatment for COVID-19, discuss research design and some explanations for why false-positive results have emerged, and argue that excessive hypothesis testing could explain the conflicting results even if no other methodological issues such as poor research design troubled HCQ repurposing trials. In Sect. 3, we continue the case study of HCQ but focus on analyzing mechanistic evidence. First, we review the laboratory results that had motivated the repurposing attempt and later negative mechanistic evidence that has conclusively shown that HCQ cannot be an effective therapy. Second, we draw a lesson from our case study and argue that negative mechanistic evidence is more reliable than laboratory results showing the drug’s efficacy. Furthermore, we claim that if more emphasis has been put on the role of mechanistic evidence and its quality, then the positive results reported by clinical trials could be interpreted as spurious earlier. Section 5 concludes.

## The problem of excessive hypothesis testing

In this section, we argue that false-positive results reported by the trials repurposing drugs for COVID-19 may have emerged due to the problem of multiple comparisons (base rate fallacy). It denotes a situation when conducting more than one statistical inference from the same or dependent datasets. It is well known that, in such cases, p-values are underestimated, and type-I errors (rejecting the null hypothesis of no effect when it is true) happen more often than could be expected (Dunn, 1961; Tukey, 1953). In Sect. 2.1., we apply statistical arguments regarding spurious findings due to multiple comparisons to the field of COVID-19 drug-repurposing trials. In Sect. 2.2., we analyze the repurposing attempts of hydroxychloroquine to show that researchers do not take into account the number of trials testing the same hypotheses and discuss some plausible explanations.

### Estimating the number of false-positive results

It is well known that false-positive results can also emerge when several research teams address the same research question (Benjamini & Hochberg, 1995; Tannock, 1996), and we argue that the field of drug repurposing for COVID-19 is susceptible to this problem. In unfavorable circumstances, multiple comparisons can drastically impede true inferences. For example, Colhoun et al. (2003) estimate that as much as 95% of genome-wide research report false-positive results. False-positive results are also more frequent than expected in clinical trials (Cleophas & Zwinderman, 2006), which impedes drug approval (van Ravenzwaaij & Ioannidis, 2019). Ioannidis (2005) show that false discovery rate (the ratio of false-positive to all positive results) depends positively on the number of tested relationships and negatively on the prior probability of tested hypotheses and the number of effective therapies. Unfortunately, the field of drug repurposing for COVID-19 is populated by numerous trials (¼ include less than 1000 participants) that are too small to report significant differences in mortality (Kimmel et al., 2020). Underpowered RCTs are more likely to report false-positive findings (Christley, 2010).

False-positive results can emerge due to random allocation of patients to treatment and control arms and individual differences in disease progression. Unwarranted claims regarding treatment efficacy are most likely to emerge in cases when the natural course of the disease is such that most patients’ health improves over time (Stegenga, 2018). In those cases, observing the improvements of patients in the treatment group can erroneously be ascribed to treatment if the control group accidentally includes a larger proportion of cases that deteriorate. A majority of COVID-19 cases are moderate. About 10–20% develop into a severe disease requiring hospitalization (Alexandrova et al., 2021). Therefore, researchers conducting RCTs can ascribe random differences in outcomes between treatment and control arms arising from sampling to the intervention despite the treatment has no effect on the course of the disease.

Considering the dominance of the frequentist approach, to account for the random changes in outcomes, only statistically significant results (i.e., unlikely to emerge by chance alone) are taken as evidence for treatment’s efficacy. The statistical significance threshold $\mathrm{\alpha }$ is usually set at the level of 0.05 (more conservative thresholds of 0.01 or 0.001 are sometimes employed). Under normal circumstances, not only statistical significance but also clinical relevance of the size of treatment effect is taken into account (Kieser et al., 2013). However, the case of COVID-19 was exceptional in the lack of any treatments, with the exception for drugs that are known to alleviate specific symptoms (e.g., fever or inflammation). Therefore, any reduction in mortality or disease progression would be considered a clinically beneficial effect, and hence we can focus exclusively on the problem of statistical hypothesis testing.

The threshold of statistical significance $\mathrm{\alpha }$ also denotes the probability of type-I error (false-positive result). In case $\mathrm{\alpha }=0.05$ (on average), one in twenty RCTs for which the null hypothesis is true will report a false-positive result. This allows for estimating the mathematical expectancy of the number of false-positive results for a group of studies. Assuming that the drug repurposing for COVID-19 is the null field (i.e., no tested compounds make any difference in comparison to their controls), the number of false positives ($\mathrm{FP}$) is given by (Tannock, 1996):

$$\mathrm{FP}=\mathrm{n}*\mathrm{\alpha },$$

where $\mathrm{FP}$—number of studies reporting (false-) positive results; $\mathrm{n}$—number of null studies; $\mathrm{\alpha }$—the threshold of statistical significance/type-I error probability $\alpha \, = \,{\text{Pr}}\,\left( {{\text{rejecting}}\,{\text{H}}_{0} |{\text{H}}_{0} \,{\text{is}}\,{\text{true}}} \right)$.

**Fig. 1:**
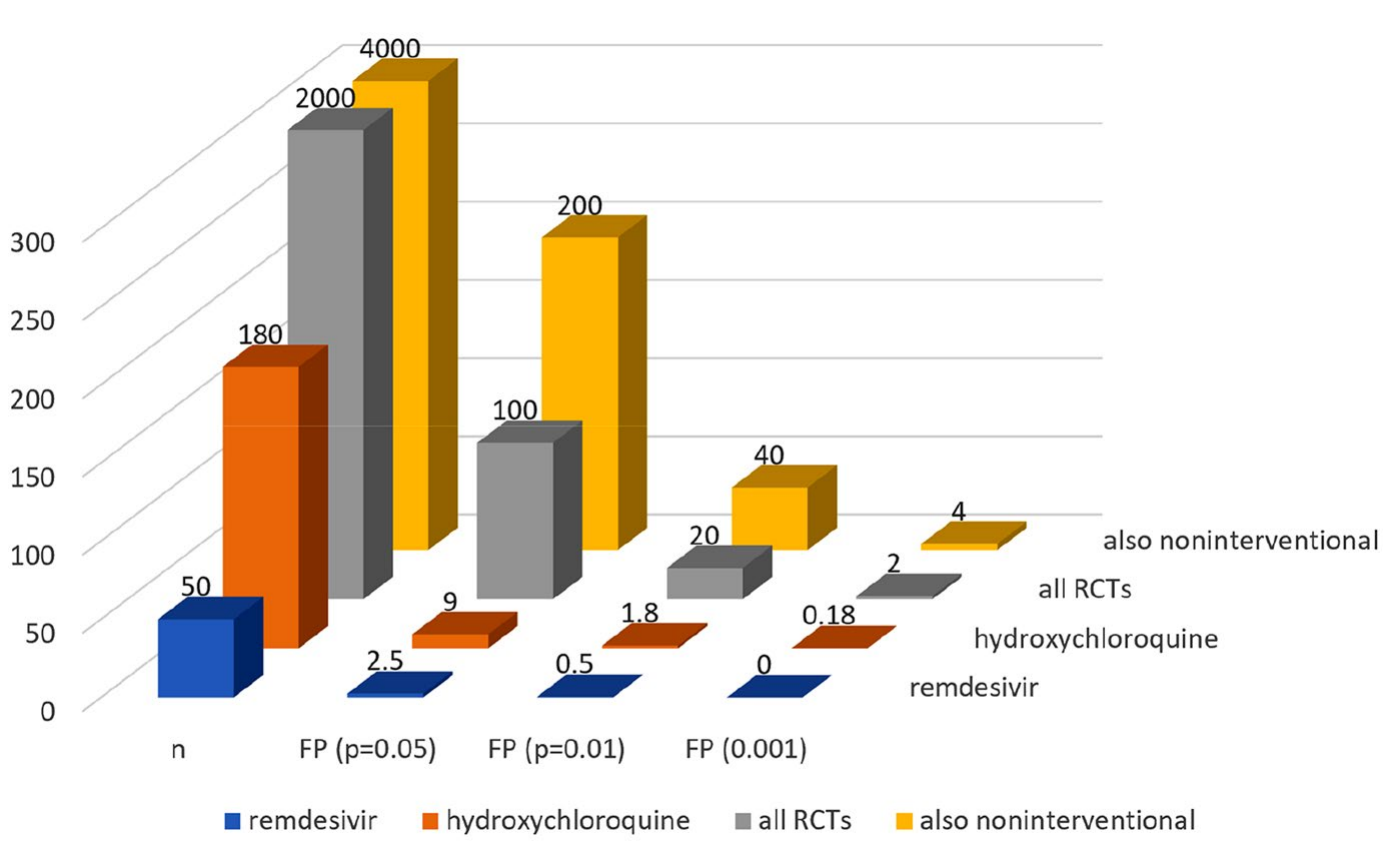
The expected number of false-positive results (own calculation)

Suppose this pessimistic scenario is accurate and none of the candidates for repurposing tested in more than two thousand active RCTs is effective. In that case, the number of false positives can be expected to be as high as 100 (for $\mathrm{\alpha }=0.05$). In a similar vein, one can estimate the expected number of false-positive results for the studies of individual drugs and for different thresholds $\mathrm{\alpha }$, see Fig. 1.

In addition, one can calculate the probability of obtaining at least one false-positive result (Dunn, 1961):

$$\mathrm{P}(\mathrm{FP}\ge 1)=1-{(1-\mathrm{\alpha })}^{\mathrm{n}}$$

**Fig. 2:**
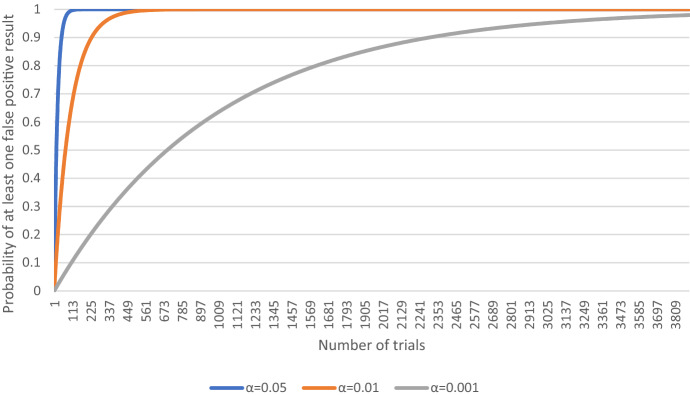
The probability of obtaining at least one false positive result $\mathrm{P}(\mathrm{FP}\ge 1)$ (own calculation)

The formula shows that for as little as 283 trials (compared to the number of studies in the field of drug repurposing for COVID-19), the chances of *not* obtaining at least one false-positive result are one to billion (1:1,000,000) when $\mathrm{\alpha }=0.05$, see Fig. 2.

Still, it is possible that some treatments will turn out to be genuinely effective and some RCTs will report true positive ($\mathrm{TP}$) results. In that case, to calculate the False Positive Report Probability ($\mathrm{FPRP}$), i.e., the probability that a positive result is a false positive, the number of true positive results needs to be extracted from the number of all studies:

$$\mathrm{n}=\mathrm{N}-\mathrm{N}*\uppi,$$

where $\mathrm{N}$—number of all studies; $\uppi$—the ratio of genuinely effective therapies to all tested therapies.

Assuming that the power of each study equals 1, i.e., the probability of accepting the null hypothesis when it is in fact true (type-II error) equals zero ($\upbeta$ =0), one can calculate $\mathrm{FPRP}$ (this idealizing assumption will be lifted later):

$$\mathrm{FPRP}=\frac{\mathrm{n}*\mathrm{\alpha }}{\mathrm{N}*\uppi }$$

**Fig. 3:**
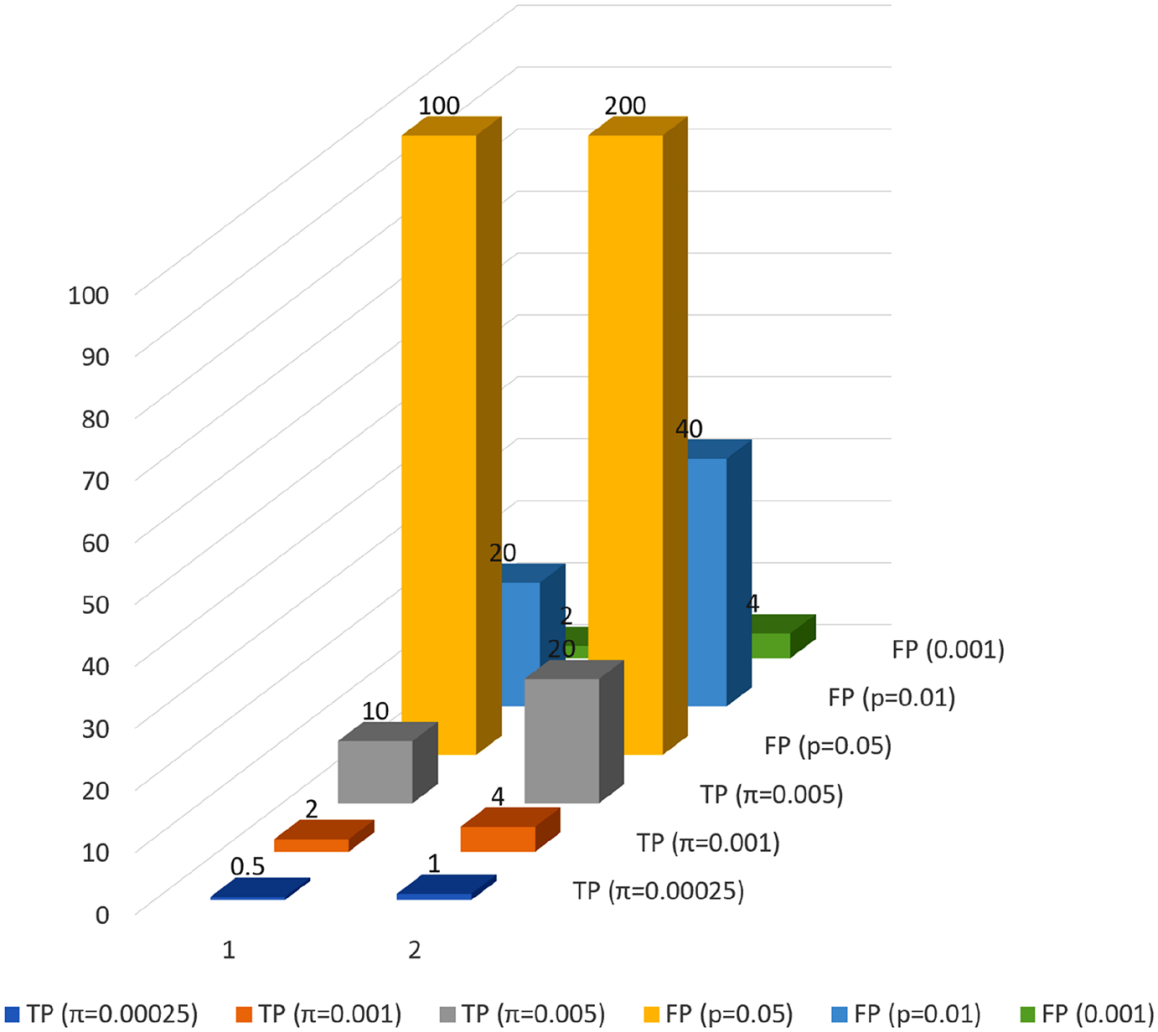
A comparison of expected numbers of true-positive to false-positive results for all RCTs (1) and all studies, including non-interventional studies (2)

Assessing the number of true positive results proves difficult, but a range of plausible values for π can be indicated. We estimate the expected number of true-positive results for several plausible values of $\uppi$: 0.00025; 0.001; 0.005; 0.03, see Fig. 3. Considering the overall number of clinical studies $\mathrm{N}$, these values can be interpreted as assumptions that there are, respectively, 0–1; 2–4; 10–20; 60–120 effective treatments under investigation. The lower bounds of the intervals are calculated by multiplying the number of RCTs assessing the efficacy of repurposed drugs against COVID-19 by $\uppi$ and the upper bounds are estimated for all clinical trials, including non-interventional studies.

Finally, we analyze $\mathrm{FPRP}$ under the assumption that some studies testing genuinely effective drugs will report (false) negative results. In that case, the probability that a positive result has been obtained despite ineffective treatment is higher and given by the following formula (Wacholder et al., 2004):

$$\mathrm{FPRP}=\frac{\mathrm{\alpha }(1-\uppi )}{\mathrm{\alpha }\left(1-\uppi \right)+\uppi (1-\upbeta )},$$

where $\uppi$—prior probability that ${\mathrm{H}}_{1}$ is true/the ratio of effective to non-effective drugs; $1-\upbeta$—statistical power; $1 - \beta \, = \,{\text{Pr}}\,\left( {{\text{rejecting}}\,{\text{H}}_{0} |{\text{H}}_{1} \,{\text{is}}\,{\text{true}}} \right)$; FPRP—False Positive Report Probability ${\text{Pr}}\left( {{\text{H}}_{0} \,{\text{is}}\,{\text{true}}|{\text{H}}_{0} \,{\text{was}}\,{\text{rejected}}} \right)$ .

**Fig. 4:**
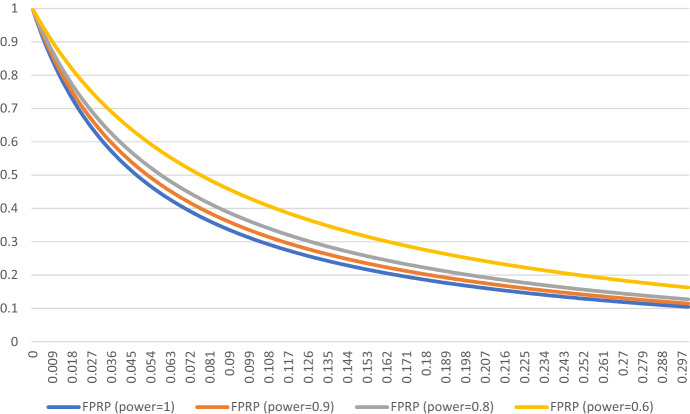
The simulation of FPRP for the field of drug repurposing for COVID-19 for $\pi \in (0-0.3)$ *and* $\beta \in (0.6, 0.8, 0.9, 1)$

Our sensitivity analysis (Fig. 4) shows that for the values of π meaningful in the context of drug repurposing for COVID-19, the statistical-significance threshold values standardly used in clinical research, and the range of expected power of studies, false-positive results dominate positive outcomes. In particular, the results show that the studies in the field of drug repurposing for COVID-19 are more likely to report false-positive results than true-positive findings. This conclusion has been obtained despite not taking into account such factors as poor research design, bias, and questionable research practices that may additionally raise the number of studies reporting positive outcomes.

### The case of hydroxychloroquine

In order to show that false-positive results are not only a statistical possibility but have indeed been reported by some COVID-19 drug repurposing studies and may be accountable for the changes in clinical guidance, we discuss the empirical literature assessing the efficacy of hydroxychloroquine (HCQ) as treatments for COVID-19. Our take on the positive results of HCQ repurposing trials does not exclude other reasons that may have played a role since biases resulting from poor research design or scientific misconduct could also shape the results of some studies. Due to the number of clinical trials, the use of HCQ in clinical practice (with the Food and Drug Administration’s emergency use authorization being the prime example), and heated debates about the treatment’s purported efficacy, HCQ is a paradigmatic example of the problem we approach in the paper.

The hype for hydroxychloroquine had been started by a few positive results of observational studies and was fueled by the urgent need for any therapy in the face of the pandemic. The simplified view on the chronological order of published results is that first positive results of small and biased studies [e.g., the randomized controlled trial from China (Chen et al., 2020) and a small observational study from France (Gautret et al., 2020)] were later contradicted by larger and methodologically-sounder RCTs (e.g., Horby et al., 2020) (see Singh et al., 2020). For instance, the note explaining the reasons for withdrawing the EUA by FDA explains that “[e]arlier observations of decreased viral shedding with HCQ or CQ treatment have not been consistently replicated and recent data from a randomized controlled trial assessing the probability of negative conversion showed no difference between HCQ and standard of care alone.” (Hinton, 2020, p. 2).

In contrast to this simplified view, some larger studies published later have also reported positive results (e.g., Arshad et al., 2020). These positive results have been primarily explained away by reference to poorer methodological quality and bias of studies reporting HCQ efficacy. For example, Fiolet et al., (2021a, 2021b) concluded that study’ risk of bias and reported efficacy of HCQ are positively related so that more biased studies are more likely to report clinically significant improvements. Singh et al. (2020) also supported this conclusion and delivered an in-depth discussion of the two studies that started the hype for HCQ. The researchers argued that the first RCT reporting positive result conducted by Chen et al. (2020) in Wuhan disobeyed study protocol by reporting results for only one dosage and was stopped prematurely. Also, the small observational study from France (Gautret et al., 2020) failed at obeying the study protocol by delivering results of assessment made on day 6 instead of 10 and excluding 6 patients from the treatment group due to their transfer to an intensive care unit (ICU). Furthermore, Rosendaal (2020) pointed out several flaws in the design and conduct of the Gautret et al. (2020) study: misinterpreting statistically insignificant differences in baseline characteristics between treatment and control groups as evidence for no difference between the groups, insufficient comparison of the treatment and control groups, questionable choice of endpoint (viral load in nasopharyngeal swabs), and a breach of the clinical trial protocol. The commentator concluded that excluding patients that deteriorated introduced selection bias to the analysis and, considering all flaws, the study of Gautret et al. (2020) should only be interpreted as a case series. Furthermore, Rosendaal (2020) pointed out that the natural course of disease explains the results of the treatment group.

All these methodological issues undermining the soundness of design and conduct of these studies could bias efficacy estimates upwards. However, the studies reporting negative results have also been criticized for delivering HCQ too late in the course of the disease (after SARS-CoV-2 replicated in patients) or used doses that cause side effects and increase mortality in treatments arms. Million et al. (2020) explained the inferior design of those studies with vested interests of the pharmaceutical industry that would benefit from developing new compounds but not from cheap generic drugs: “it seems that there is a competition between low-cost generic medications that are potentially effective (…) and very expensive new drugs that are not yet approved, implying financial and organizational issues, stakeholders expectations and administrative/policy complexity.”

Even though vested interests and inferior design of some studies seems a plausible explanation of the conflicting results at first glance, detailed analysis of the designs shows that methodological problems populate studies reporting both positive and negative results. For example, it is true that antiviral agents that target replication mechanism are only effective at earlier stages of the disease and first studies tested the efficacy of HCQ in hospitalized patients, but more recent reports showed that HCQ does not treat early COVID-19 too. For example, Skipper and Boulware (2021) conducted an RCT assessing the efficacy of HCQ in outpatients suffering from COVID-19. The investigators planned to use hospitalization or death as the primary endpoint but exchanged it with health assessment on days 5, 10, and 14 due to a worry regarding statistical power. The use of the novel internet-based design and delivering treatments or placebo by post allowed for recruiting patients early after symptom onset. Nevertheless, despite the use of a sufficient sample size of 491 patients (of which 423 contributed to the result) and HCQ dosing sufficient to achieve maximal effective concentrations for SARS-Cov-2 inhibition, the treatment did not have a statistically significant effect on the course of the disease.

This negative result can be contrasted with the Henry Ford Study (Arshad et al., 2020), an even larger study that showed that HCQ alone and in the joint therapy with azithromycin (AZA) is useful for treating COVID-19: the former treatment reduces the hazard ratio of death by 66% and the latter by 71% (p < 0.001). This case–control study has been conducted on the group of 2541 patients with RT-PCR tests confirming infection with SARS-CoV-2 randomized into three treatment arms (HCQ, AZA, HCQ + AZA). Even though RCTs are usually considered as a superior source of evidence in comparison to cohort and case–control studies, the standard view may not be appropriate in the case of literature assessing the efficacy of HCQ as a treatment for COVID-19. The reason is that, with some exceptions (e.g., Horby et al., 2020), the majority of RCTs (e.g., Cavalcanti et al., 2020; Self et al., 2020) used outcomes that are susceptible to bias such as viral clearance, clinical improvement, and viral load instead of mortality. This is problematic because even if HCQ shortened patients’ viral shedding time, the question of how it translates into mortality or improves the course of disease remains uncertain (Paul, 2021). In contrast to RCTs, more observational studies used mortality as the primary outcome what improved their reliability due to larger samples: given that mortality is a considerably rare outcome in COVID-19, the sample size needs to be much larger than the number of participants in most RCTs.

The positive results have been obtained despite controlling for known prognostic factors and the Henry Ford Study is considered methodologically sound and justifying the use of HCQ in clinical practice (e.g., Somberg, 2020). Similar results have been obtained by, for example, the investigators from Mount Sinai hospitals in New York (Wang et al., 2020) and researchers from France (Million et al., 2020). But there are also observational studies that reported that HCQ has no effect on mortality or other outcomes. Interestingly, negative results have been reported by both large and methodologically sound studies and small and at the risk of bias. For example, Geleris et al., (2020) concluded on the basis of analyzing 1446 consecutive patients with COVID-19 at the emergency department of New York Presbyterian Hospital that HCQ has no effect on the risk of intubation or death. Another negative result reported by Mahévas et al. (2020) is based on the sample size of only 173 patients and concerned with the risk of transfer to the intensive care unit.

Arshad et al. (2020) explained their positive results with using standard dosing of HCQ. It is also possible that excluding patients susceptible to the cardiovascular side effects of HCQ with electrolyte analysis and EKG improves the prognosis of the treatment arm. However, some studies reporting positive results employed broader inclusion criteria and nevertheless reported positive outcomes. For instance, Chen et al. (2020) randomized 62 patients fulfilling the inclusion criteria specifying mild COVID-19 only (i.e., adults, SARS-CoV-2 infection confirmed with RT-PCR, chest CT confirming pneumonia, and limited deterioration in lung function). Despite not excluding patients susceptible to HCQ cardiovascular harm, pneumonia improved in 80.6% of the treatment group and only 54.8% of the control group.

The studies repurposing HCQ for COVID-19 are heterogeneous in regard to methodological soundness and the risk of bias. And while some studies may have reported spurious positive results due to poor design or (possibly unconscious) scientific misconduct, the view that spurious findings have only been reported by methodologically unsound studies is not warranted. As we argued in Sect. 2.1., false-positive results can also emerge due to random allocation of patients to treatment and control arms and individual differences in disease progression. The view that false-positive results emerge due to excessive hypothesis testing is even more plausible considering that some RCTs assessing the efficacy of HCQ likely remain unpublished: in a commentary published as a preprint, Gao et al. (2020) mentioned 11 RCTs assessing the efficacy of HCQ being underway in China but none of these studies (with the exception for Chen et al., 2020) was published until now, what creates the base-rate fallacy (see Bird, 2020). Nevertheless, even considering only the number of published studies assessing the efficacy of HCQ can account for positive results. The most recent systematic review identified 11 RCTs and 1241 observational studies (Eze et al., 2021). Considering only published studies leads to the conclusion that one should expect approximately one false-positive result reported by an RCT and 62 by observational studies (see Sect. 2.1).

Despite these characteristics of the field of studies repurposing HCQ for COVID-19, most studies do not control for base rate fallacy and those exceptional cases that control for the number of statistical comparisons and lower the significance thresholds accordingly, take into account only the number of trial arms and not the base rate of the whole field. The question of whether an alpha correction for a field’s false discovery rate should be introduced at the level of individual studies remains debatable. Rubin (2021) argued recently that “if researchers make a decision about a joint null hypothesis after rejecting at least one (and not all) constituent null hypotheses, then an alpha adjustment is necessary.” (p. 26). And this is precisely the situation of inferences drawn from repurposing studies: due to the extraordinary circumstances, clinical decisions have been made based on single positive results. Here, we need to highlight that the context of the pandemic is exceptional, and alpa correction is less required when the standard practice of drug approval relying on positive results of two phase-III independent studies is obeyed. However, applying methods such as Bonferroni correction is problematic in practice and methodologically dubious. For example, Ioannidis (2005) observed that “usually it is impossible to decipher how much data dredging by the reporting authors or other research teams has preceded a reported research finding”. Similarly, Perneger (1998) pointed out that estimating the number of statistical tests requiring adjustment is difficult and may be impossible: “Most proponents of the Bonferroni method would count at least all the statistical tests in a given report as a basis for adjusting P values. But how about tests that were performed, but not published, or tests published in other papers based on the same study? If several papers are planned, should future ones be accounted for in the first publication? Should we worry about error rates related to an investigator—taking the number of tests he or she has done in their lifetime into consideration—or error rates related to journals?”. Furthermore, adjusting for multiple comparisons reduces the chances of discovering true effects, i.e., it increases the type-II error rate (Perneger, 1998; Rothman, 1990). This is particularly true of the field of COVID-19 drug repurposing trials due to the number of statistical tests (see Sect. 2.1.). Bonferroni correction and other approaches controlling for family-wise error rates are known to be too conservative and sacrifice power. Using the less conservative methods of controlling for false discovery rate, such as the BH procedure may be a more viable option (Benjamini & Hochberg, 1995). However, deciding on the alpha level always boils down to the choice between type-I and type-II error rates. The urgent need for finding a cure (especially at the start of the pandemic) is a good argument for not applying the correction.

Systematic review with meta-analysis allows for obtaining an estimate of average treatment effect more accurate than the results of individual studies (Oxford Centre for Evidence-Based Medicine, 2009; National Institute of Health and Care Excellence, 2014) and is standardly used to amalgamate evidence from clinical trials. Meta-analysis can be helpful in detecting false-positive results because they can only account for a small fraction of results in a field. Unfortunately, the conclusions of the meta-analyses of HCQ repurposing studies are also conflicting: Million et al. (2020) concluded that HCQ is effective, which contradicts the results of other systematic reviews (Eze et al., 2021; Fiolet et al., 2021a, 2021b; Singh et al., 2020). The investigators obtained the positive efficacy estimate by dismissing the results of observational studies and focusing on a chosen subgroup of interventional studies. They justified these choices by claiming that retrospective studies of health records lack detailed dosage information and are of low quality and therefore should not inform therapeutic decisions. Due to the exclusion of a substantive number of studies, the positive assessment of HCQ’s efficacy of Million et al. (2020) is based on three out of four RCTs reporting positive results. To justify the exclusion of one RCT, the researchers pointed out that antiviral treatments have a narrow therapeutic window and hypothesized that conflicting results have emerged because some studies delivered HCQ treatment after SARS-CoV-2 replicated in patients or used doses that cause side effects and increase mortality in treatment arms.

While the meta-analysis claiming HCQ efficacy may be politically driven and rely on excluding studies reporting negative results [see (Stegenga, 2011) for the discussion of the malleability of meta-analysis], this can only be established in the light of other meta-analyses (Eze et al., 2021; Fiolet et al., 2021a, 2021b), and large and well-designed trials (e.g., Horby et al., 2020). Below, we argue that applying the advice of evidential pluralism to use both difference-making and mechanistic evidence to assess efficacy and harm in medicine is beneficial and could help draw the correct conclusion from conflicting difference-making results of clinical studies sooner. In particular, we show that positive mechanistic evidence for HCQ was of low quality and novel results showed that HCQ is unable to prevent SARS-CoV-2 from replicating in human lungs.

## Using mechanistic evidence to assess the efficacy of repurposing candidates

Above, we have argued that the number of clinical trials repurposing existing drugs as treatments for COVID-19 can lead to false-positive results. In this section, we use the example of HCQ to argue that negative mechanistic evidence jointly with weak difference-making evidence suffices to conclude that the repurposing candidate is not effective. Furthermore, we claim that if more emphasis had been put by decision-makers on obtaining high-quality mechanistic evidence, then HCQ would probably not enter clinical practice based on spurious results of clinical trials. We also argue that negative mechanistic evidence is more reliable than positive mechanistic evidence due to possible interactions of the drug’s mechanism of action in vivo (Sect. 3.2.).

### Negative mechanistic evidence against HCQ efficacy

Our argument is inspired by the observation that, despite some spectacular examples of successful repurposing attempts such as Viagra (Ashburn & Thor, 2004; Neuberger et al., 2019), most compounds target precise biological processes and are ineffective beyond their domain. This makes the process of drug repurposing marked with failure. For example, amantadine targets only influenza virus A and is ineffective for influenza virus B (Jackson et al., 2011) despite a high degree of similarity between the two pathogens. Neuberg et al. (2019) analyzed the complete clinical development history of 834 drug candidates that entered clinical trials between 1980 and 2012. They discovered that less than 2% of them were ultimately launched in a therapeutic area other than the one they were developed for. The success rate is higher for drugs repurposed within the same therapeutic area, e.g., the drugs developed for breast cancer have been successfully repurposed for ovarian cancer. The low success rate of the repurposing studies has been observed under ordinary circumstances, when the process of selecting candidates for repurposing lasts, on average, about 2 years (Ashburn & Thor, 2004). In the case of drug repurposing for COVID-19, this process has been accelerated and many trials had been started before convincing mechanistic evidence for drugs’ efficacy was available. This acceleration is understandable considering the extraordinary circumstances of the pandemic and no treatment available for deteriorating patients.

HCQ was suggested as one of the early candidates for treating COVID-19 patients (Liu et al., 2020; Yao et al., 2020). HCQ is a widely used and relatively safe anti-malaria drug. Hydroxychloroquine is an analog of chloroquine that is safe and more popular because it is less likely to interact with other drugs. In recent years, chloroquine and HCQ has been shown in vitro to have antiviral, anticancer, and antifungal properties (Alani et al., 2020; Rolain et al., 2007). Therefore, it is not surprising that SARS-CoV-2 was suggested as another potential target. The suggestion results from laboratory research whereby HCQ and chloroquine were shown to inhibit the ability of SARS-CoV-2 to infect African green monkey kidney Vero cells (Liu et al., 2020; Yao et al., 2020). These results have been used as a reason for starting more than 1000 clinical studies, some of which have been prematurely terminated.

Despite some positive outcomes that, in the light of our analysis, can be interpreted as false positives, the larger and more conclusive studies have reported insignificant effect of hydroxychloroquine on the course of COVID-19 (Boulware et al., 2020; Mitjà et al., 2020). This might be surprising considering that the mechanistic evidence from the in vitro research supporting the efficacy of HCQ was considerably well justified. However, new negative mechanistic evidence had emerged more recently. Hoffman et al. (2020) discovered the exact mechanism blocking the replication of SARS-CoV-2 in African green monkey kidney Vero cells. This mechanism remained unknown at the time when clinical trials of HCQ were started and HCQ authorized for treating COVID-19. SARS-CoV-2 can enter cells by two different mechanisms. First, the SARS-CoV-2 spike protein attaches to the ACE2 receptor and inserts its genetic material into the cell. Second, the virus is absorbed into endosomes (a part of the endocytic membrane transport pathway). Depending on the cell type, the enzymes involved in these mechanisms might be different. Some, like kidney cells, need an enzyme called cathepsin L for the virus to infect them successfully. Others, like lung cells, need an enzyme called TMPRSS2 (on the cell surface). Cathepsin L requires an acidic environment to function and allows the virus to infect the cell while TMPRSS2 does not. HCQ increases the endosomal pH of cells and inhibits viruses that depend on low pH for cell entry (Rolain et al., 2007). Hoffman et al. (2020) showed that, in the green monkey kidney cells, HCQ decreases the acidity, disables the cathepsin L enzyme, and blocks the virus from infecting the kidney cells. In human lung cells, which have deficient levels of cathepsin L enzyme, the virus uses the enzyme TMPRSS2 to infect the cells. Given that the enzyme is not controlled by acidity, HCQ is unable to block SARS-CoV-2 from infecting the lung cells or stop the virus from replicating.

This novel negative mechanistic evidence suggests that HCQ cannot be an effective treatment that targets the replication of SARS-CoV-2 in human lung cells. This sheds new light on the difference-making evidence stemming from clinical trials. Considering the poor quality of difference-making evidence caused by excessive hypothesis testing (see Sect. 2.2.) and moderate or high risk of bias in HCQ repurposing trials (Mazhar et al., 2020), jointly with new negative mechanistic evidence, allows for concluding that HCQ is not an effective treatment for COVID-19. This poses the question of whether it was justified to trust previous in vitro results obtained in African green monkey kidney Vero cells. Below, we argue that the example of HCQ shows that the endeavor of drug repurposing for COVID-19 was not relying on high-quality mechanistic evidence, but in vitro research was directly extrapolated into humans with disregard for the mechanism by which HCQ interferes with SARS-CoV-2 replication in the tube.

### Mechanistic evidence and the results of drug repurposing trials

The urgent need for having an effective therapy for the disease explains why pharmaceutical treatments of COVID-19 have often been approved by medical institutions, without the evidence that would be required under ordinary circumstances (Hofmann, 2020). Most of the therapies that have been investigated and authorized by drug agencies are repurposed drugs. Even though drug repurposing seems to be a very promising approach to find a treatment for COVID-19, we have shown that the excessive number of clinical trials lead to obtaining false-positive results. This poses the question of how to assess the efficacy of repurposing candidates given that the evidence stemming from drug repurposing clinical trials is of low quality. While starting clinical trials only after sufficient mechanistic evidence for repurposing candidates’ efficacy and designing large multi-center studies would diminish the likelihood of obtaining false-positive results, these solutions are infeasible in public health emergencies such as the pandemic of SARS-CoV-2. Under such unusual circumstances, researchers and clinicians try independently to find an effective cure, and drug agencies and physicians considering off-label use of existing therapies are the audience for the drug repurposing empirical literature and they have limited impact on the quality and design of the studies.

Statisticians have developed many methods of controlling for multiple comparisons and false discovery rate in a field (Glickman et al., 2014; Sedgwick, 2012) but using this approach in practice is problematic for the reasons discussed in Sect. 2.2. Another approach to telling false-positive from true positive findings would be to analyze effect sizes. For example, a reduction of hospitalization duration by just 1 day can be considered as a clinically insignificant effect that may have arisen by chance alone, especially if no change in mortality is observed. In contrast, if a tested compound would drastically reduce mortality, then it could be considered a genuinely positive result. Extraordinary effect sizes have previously been observed in cases when effective treatments targeting virus’ molecular mechanisms were developed. For example, antiretroviral therapy is considered one of the most outstanding achievements of modern medicine (Laskey & Siliciano, 2014). However, the lack of any therapeutic options makes decision-makers more willing to authorize the use of treatments that show any effect, even if it is clinically insignificant.

For these reasons, we argue that supplementing weak difference-making evidence with either mechanistic evidence for the drug’s efficacy against COVID-19 or negative mechanistic evidence can be helpful to draw a plausible conclusion. In particular, we argue that negative mechanistic evidence (i.e., mechanistic evidence that explains why a treatment cannot be effective) can be useful and is more believable than mechanistic evidence for treatment efficacy. Our stance results from the belief that high-quality mechanistic evidence in favor of a drug’s efficacy can support weak difference-making evidence and they, jointly, may warrant authorizing a treatment. This approach can speed up the assessment process as waiting for the results of high-quality RCTs or meta-analyses of interventional studies is not needed. But negative mechanistic evidence can also outweigh the results of clinical trials in cases when difference-making evidence is of low quality due to, for example, base-rate fallacy. However, negative mechanistic evidence is, all else being equal, more believable because there are many mechanisms operating in vivo and interacting with each other and therefore showing that a mechanism by which a drug acts is screened off or not working in the target is more plausible than results describing a treatment’s mechanism, especially if the cell lines used in in vitro research are not the same as those targeted by the treatment under consideration. We have used the example of HCQ to argue that difference-making evidence is of low quality because of the high likelihood of false-positive results. Here, we claim that supporting decisions regarding the authorization of HCQ as a treatment for COVID-19 with an in-depth assessment of the quality of mechanistic evidence would save FDA from authorizing the use of an ineffective treatment. However, we believe that our approach is applicable to other treatments.

Initial in vitro studies of HCQ efficacy had been conducted on the African green monkey kidney Vero cells (Liu et al., 2020; Yao et al., 2020) and do not describe the mechanism by which HCQ operates. Instead, in vitro results have been directly extrapolated into humans to justify starting clinical trials and explain (false) positive results. Animal and in vitro studies are a standard way of evaluating drugs’ efficacy when new compounds are developed or existing therapies are considered repurposing candidates. Drugs that are efficacious in one species may not necessarily have the same effect in different organisms due to their mechanism of action interactions or diseases’ different mechanism. African green monkey seems to be a good candidate for the in vitro and animal phase of the repurposing process, as they are primates and so they share a lot of properties with humans. For these reasons, if a treatment is efficacious in African green monkeys, we might expect it to be efficacious in humans as well. However, there is a more important mismatch: the initial studies of HCQ efficacy were conducted in the kidney Vero cells of African green monkeys. Considering the results of Hoffman et al. (2020), HCQ would likely turned out to be ineffective in treating COVID-19 in African green monkeys if animal models were used in the process of drug repurposing because SARS-CoV-2 replicates mainly in the lungs of infected animals (Monteil et al., 2020). Taking this into account, the focus of in vitro research on a different cell lineage without a further justification of why HCQ should also work in lung cells is problematic and should raise suspicion regarding positive in vitro results.

And, indeed, if we look at further mechanistic studies of HCQ then our worries are reinforced. Later, Hoffmann et al. (2020) proved that HCQ is unable to block the replication process of SARS-CoV-2 because the virus enters human lung cells differently. These results were published later than FDA published its emergency use authorization. However, at that time, in vitro results supporting HCQ efficacy were obtained only on the kidney cells. This raises suspicion because kidney cells may not be sufficiently similar to lung cells to justify extrapolation without additional evidence. Furthermore, no studies warranted that HCQ interferes with SARS-CoV-2 replication process in lung cells in the same way as it does in kidney cells. This makes the mechanistic evidence for HCQ efficacy from the tube of low quality and does not lend support to using HCQ in clinical practice as a treatment for COVID-19. If the quality of mechanistic evidence for HCQ efficacy had been assessed earlier, FDA could probably refrain from the emergency use authorization and would not have to revoke it after new evidence emerged. As Stefan Pöhlmann (2020) pointed out in the press release discussing their results: “[t]his means that in future tests of potential COVID-19 drugs, care should be taken that relevant cell lines are used for the investigations in order not to waste unnecessary time and resources in our search for effective COVID-19 therapeutics”.

The case of HCQ shows that one should not only seek any sort of mechanistic evidence but also assert its high quality and no flaws in the reasoning from mechanisms to treatment’s efficacy. Some approaches to assessing the quality of evidence for mechanisms and reasoning from mechanisms have been developed so far (see Parkinnen et al., 2018). Our analysis suggests another factor that should be considered. The most trustworthy evidence stems from studies conducted on cell lineages that are the same as those building a treatment’s target. The less similar are cells studied in vitro to its target, the lower is the quality of obtained evidence, and our case study has demonstrated that mechanistic evidence stemming from inappropriately designed studies can easily turn out spurious.

However, reasoning from mechanistic evidence is always fallible because many mechanisms are operating in living organisms at the same time and interacting with each other. These interactions among different mechanisms cannot be traced in vitro (see Aronson et al., 2021) Therefore, even accurate knowledge of one mechanism is insufficient for establishing causality (Russo & Williamson, 2007) or predicting the effects of pharmaceutical treatment. The example of HCQ also shows that there is an asymmetry in the credibility of mechanistic reasoning for a treatment’s efficacy and against it (i.e., negative mechanistic evidence). In other words, it is preferable (from the perspective of drawing sound conclusions from the body of empirical literature) to show that a drug does not work in adequate cell lineages in vitro instead of showing that it works. Negative mechanistic evidence can help in discriminating repurposing candidates that are unlikely to be effective but it can also be used to shed light on the reasons why clinical studies have reported conflicting results as it is doubtful that a compound that does not work in cell cultures works in organisms. What follows, if difference-making evidence stemming from clinical trials is of low quality or conflicting due to base rate fallacy in the case of COVID-19 trials or possible bias, one can use negative mechanistic evidence to exclude causality but positive in vitro results only weakly support efficacy claims. The negative mechanistic evidence alone should inspire caution in interpreting early difference-making evidence regarding efficacy of hydroxychloroquine.

This discussion about the role of mechanistic evidence, with emphasis on the negative one, should not obfuscate our main message. We are not arguing that mechanistic evidence should be considered more reliable than difference-making evidence stemming from clinical trials but claim that both types of evidence should be used jointly as they are interconnected. Despite the inter-related nature of difference-making and mechanistic evidence, according to EBM+, some research methods (such as RCTs and observational clinical studies) are more helpful in producing difference-making evidence. In contrast, others (e.g., in vitro research, screening technologies) are more suitable for inferring mechanisms (Parkkinen et al., 2018). This being said, the research methods typically used to produce difference-making evidence can also deliver evidence for mechanisms and vice versa: the designs typically delivering mechanistic evidence can also provide (possibly only weak) evidence for difference-making.

The main advantage of randomization is that RCTs can serve as a black-box tool (Howick, 2011) that supports causal conclusions even if the details of how an intervention works remain unknown. As Martinez and Teira (2020) have recently put it, “there are good reasons to randomize the allocation of treatments, in particular when there is no agreement among experimenters as to the antecedent conditions to be controlled for” (Martinez and Teira 2020). Well conducted RCTs assert that the difference between treatment and control groups can be interpreted as resulting from the intervention (Illari, 2011; La Caze, 2009). However, some specifically designed RCTs can deliver mechanistic evidence (evidence for a mechanism). For instance, Marchionni and Reijula (2019) argued that properly designed laboratory experiments in economics allow for discriminating between two plausible mechanisms producing a phenomenon and hence deliver evidence for one of them. The same can be said about RCTs in medicine. For example, one could conduct an RCT to test the mechanism of interaction between temozolomide (a drug developed for glioblastoma, a brain tumor) and the MGMT gene (Blunt, 2019). If the RCT would confirm the observational result of Hegi et al. (2005) that patients with the gene do not respond to therapy, then it would support our current (mechanistic) understanding of how the gene codes a protein that counteracts the mechanism of the drug’s action. However, most RCTs assessing efficacy rely on some mechanistic evidence (evidence from mechanisms or mechanistic reasoning) (Rocca, 2018) but observing the difference between treatment and control groups delivers difference-making evidence. The mechanistic evidence understood as features of specific mechanisms, which enters RCTs at the planning stage, emerges from basic science research (La Caze, 2011) such as in vitro and in vivo experiments, observation technologies, and analogy (Parkkinen et al., 2018, p. 78).

Another take on how RCTs could deliver mechanistic evidence is to argue that by demonstrating the link between intervention and outcome, RCTs provide evidence that some mechanism links the two. For example, a positive result showing that HCQ is an effective treatment for COVID-19 would deliver some evidence that there is a mechanism between the intervention and outcome. There are two problems with this reasoning. First, as Howick (2011, p. 34) observed, it is circular due to taking the observation of difference-making as a sign of an underlying mechanism. The reason is that accepting the mechanistic ontology equates to acknowledging that mechanisms produce causal relations, and observing such relations is a sign of an underlying mechanism. Second, even if the problem of circularity could be ignored, RCTs do not depict a hypothesized underlying mechanism. Referring to the views on what are the sources of evidence for mechanisms in medicine supports this view. Illari (2011, 2016) defined such evidence as the technologies that allow for studying entities constituting a mechanism and their interactions, in particular studies that help identify or better understand entities, their activities, and organization. Also, Parkkinen et al. (2018) do not include RCTs in research designs that deliver mechanistic evidence. To reiterate, well-conducted RCTs are very good at showing that an intervention under study leads to an observed outcome regardless of the mechanism of action. On this ground, they have been prioritized by the EBM movement.

However, if the difference-making evidence is of low quality due to either excessive hypothesis testing raising the chance for obtaining false-positive results or poor research design introducing biases, efficacy claims are less reliable and we argued, in line with the approach of EBM+, that decisions regarding treatment efficacy should additionally be supported by mechanistic reasoning. Seeing both as mutually interconnected is the best way of thinking about the dependence of mechanistic and difference-making evidence. Considering a few scenarios is worthwhile. Strong difference-making evidence stemming from well-conducted RCTs in connection with positive mechanistic evidence would reinforce one’s trust in the results of clinical trials. If strong difference-making evidence were contrasted with negative mechanistic evidence, it would make one reconsider the nature of mechanistic evidence. For instance, such a situation could be explained by the paradoxical effects caused by some therapies. However, the conclusion may differ if strong mechanistic evidence is considered jointly with weak difference-making evidence. A dose of skepticism is needed in this case as in vivo studies may not necessarily mirror the complexity of interactions in living organisms. Alternatively, strong negative mechanistic evidence combined with weak difference-making evidence suggests that the observed effect size is likely spurious, especially if bias is present or spurious findings are likely to be reported by clinical trials.

All in all, there is a continuum of possible results delivering difference-making and mechanistic evidence. It would be best to obtain strong evidence of both types to claim treatment efficacy. However, the SARS-CoV-2 pandemic, an unprecedented public health emergency, requires making decisions far from ideal circumstances. This requires using any evidence that is currently available, but it should be carefully assessed. Sometimes one type of evidence can support the other type of evidence by providing additional results. Sometimes they might stay in conflict. The case of HCQ was peculiar because neither clinical trials nor negative/positive mechanistic evidence was of high quality. Clinical decisions were based on weak difference-making evidence supported by weak mechanistic evidence. If in vitro studies were designed more carefully (e.g., having in mind the use of proper cell lineages) and the evidence stemming from clinical trials were assessed appropriately then less faith would be put in HCQ.

## Concluding remarks

Our case study of the attempt at repurposing HCQ as a treatment for COVID-19 has shown that false-positive results have emerged due to the number of clinical trials because HCQ is unable to stop SARS-CoV-2 from replicating in human lungs. One could also ask why false-positive results have emerged from the perspective of the sociology of knowledge or economics of the pharmacological industry. While further research is still needed to gather conclusive evidence, our analysis sheds light on some plausible explanations. Considering that HCQ is a cheap drug that is not protected by intellectual property law, vested interests of the industry are an unlikely factor driving starting so many trials. Therefore, researchers’ presupposition regarding HCQ efficacy seems a more likely factor influencing decisions to start clinical trials. Although we do not argue that clinical trials should not be conducted without strong mechanistic evidence for treatment’s efficacy (especially in the case of the crisis), we need to observe that excessive testing of the efficacy of one drug can possibly be used by the pharmaceutical industry instead of other questionable or fraudulent practices to spuriously show a compound’s efficacy and profit from selling stockpiled treatments. For example, remdesivir was first tested as a single therapy and later jointly with various generic drugs, what raised the chance for false-positive results to emerge. It received FDA’s emergency use authorization despite conflicting evidence, the price of over 2000 euro per therapy (Rezagholizadeh et al., 2021) and some concerns regarding the soundness of the RCT reporting its efficacy (see Holman, 2021).

Furthermore, our analysis supports the use of the program of EBM+ to assess the efficacy of repurposing candidates. If false-positive results are expected because of the number of clinical trials testing the same compounds, negative mechanistic evidence for drugs’ efficacy can influence the inferences drawn from positive results and prevent authorizing treatments based on spurious outcomes. We believe that the program may prove fruitful, especially in public health emergencies such as the SARS-CoV-2 pandemic, due to the pressing need for undertaking decisions with limited evidence. Furthermore, our case study has shown that negative mechanistic evidence is more trustworthy than in vitro results demonstrating a treatment efficacy.

Our analysis was focused on the process of drug repurposing for COVID-19. But discovering pharmaceutical treatments is not the only strategy against the COVID-19 pandemic. Developing vaccines and novel drugs are the other possibilities. The former falls beyond the scope of the paper, but the latter should be discussed briefly. Should our worries be limited solely to the case of drug repurposing or maybe as well to novel drugs explicitly developed to treat patients suffering from COVID-19? The two procedures seem to be quite different. When a new drug is being developed, several steps are required (for full comparison, see Ashburn & Thor, 2004). For instance, extensive laboratory experiments have to be carried out that map the mechanistic interactions between the drug and the organism; they include but are not limited to, in vitro studies, in vivo validation, structure-based drug design, etc. This is followed by clinical trials that include three phases. Under the usual circumstances, two positive results of phase III RCTs are required for drug approval which reduces the risk of endorsing a treatment based on false-positive results. All these steps provide a good mechanistic and statistical justification for the effectiveness of a drug. The process of developing and authorizing new drugs may be shortened due to extraordinary circumstances. If so, drug development may face the same issues.

However, we do not argue that we should refrain from clinical studies when there is insufficient mechanistic evidence in favor of certain drugs given the extraordinary circumstances. First of all, we should distinguish between the state of regular science and science doing in the state of global crisis. The current situation raises many concerns and issues, both scientific and social (Chellappoo, 2021; Mercuri, 2020). Considering the uncertainties related to the public health emergency, many decisions concerning science and its application may be made without the standard procedures. For example, the selection of repurposing candidates may diverge from the standard approach, and many more drugs may be tested due to the hope that some of them will be effective.

At the same time, we should be aware that going through shortcuts might be problematic in many ways. It might generate false hopes that might influence the way the public behaves. For instance, some people have mistakenly taken other drugs that sounded like hydroxychloroquine after hearing about it to prevent covid-19 (Samuels & Kelly, 2020). Insufficiently assessed false-positive results may lead to drug approvals that are later revoked. This leads us to what Carl Sagan once said famously in “Cosmos,” a TV program popularizing science: ‘extraordinary claims require extraordinary evidence’. We think that this phrase is particularly relevant for the field of drug repurposing for COVID-19. Efficacy claims are, without a doubt, in the current situation, extraordinary. But the evidence supporting them is not remarkable. Due to the number of repurposing attempts, efficacy claims are likely to be false-positive results. As we have argued above, considering mechanistic evidence before endorsing new treatments for COVID-19 may be useful.
